# An mRNA vaccine against rabies provides strong and durable protection in mice

**DOI:** 10.3389/fimmu.2023.1288879

**Published:** 2023-10-26

**Authors:** Miao Li, Enyue Fang, Yunpeng Wang, Leitai Shi, Jia Li, Qinhua Peng, Xingxing Li, Danhua Zhao, Xiaohui Liu, Xinyu Liu, Jingjing Liu, Hongshan Xu, Hongyu Wang, Yanqiu Huang, Ren Yang, Guangzhi Yue, Yue Suo, Xiaohong Wu, Shouchun Cao, Yuhua Li

**Affiliations:** ^1^ Department of Arbovirus Vaccines, National Institutes for Food and Drug Control, Beijing, China; ^2^ Vaccines R&D Department, Changchun Institute of Biological Products, Changchun, China; ^3^ Institute of Health Inspection and Quarantine, Chinese Academy of Inspection and Quarantine, Beijing, China

**Keywords:** rabies, rabies vaccine, RABV-G, rabies vaccine potency test, mRNA vaccine, rabies mRNA vaccine

## Abstract

**Introduction:**

Rabies is a serious public health problem worldwide for which an effective treatment method is lacking but can be prevented by vaccines. Current vaccines are produced in cell or egg cultures, which are both costly and time consuming.

**Methods:**

Here, a non-replicating mRNA vaccine (RV021) encoding the rabies virus glycoprotein was developed *in vitro*, and its immunogenicity and protective efficacy against live virus was evaluated in mice.

**Results:**

A two-dose vaccination with 1 μg of RV021 at 7-day intervals induced a protective level of neutralizing antibody that was maintained for at least 260 days. RV021 induced a robust cellular immune response that was significantly superior to that of an inactivated vaccine. Two doses of 1 μg RV021 provided full protection against challenge with CVS of 30~60-fold lethal dose, 50%. Vaccine potency testing (according to the National Institutes of Health) *in vivo* revealed that the potency of RV021 at 15 μg/dose was 7.5 IU/dose, which is substantially higher than the standard for lot release of rabies vaccines for current human use.

**Conclusion:**

The mRNA vaccine RV021 induces a strong protective immune response in mice, providing a new and promising strategy for human rabies prevention and control.

## 1 Introduction

Rabies is a highly pathogenic zoonotic disease caused by the rabies virus (RABV), a single-stranded RNA virus belonging to the genus *Lyssavirus*, family Rhabdoviridae. The disease causes approximately 59,000 deaths worldwide each year (1). Rabies is mainly transmitted by bites or scratches from an infected animal, allowing RABV entry from the wound into the host’s body. As a neurotrophic virus, RABV replicates at the wound site and enters the neuromuscular junction to finally arrive at the central nervous system. When the virus spreads to the brain and undergoes proliferation, it can finally kill the host (2, 3). Rabies is nearly 100% fatal upon onset but can be effectively prevented by rabies vaccination (4).

The RABV genome encodes five proteins: glycoprotein (G), nucleoprotein (N), matrix protein (M), phosphoprotein (P), and RNA-dependent RNA polymerase (RdRp) (5). RABV-G mediates the viral attachment to various receptors, such as the neural cell adhesion molecule (6), nicotinic acetylcholine receptor (7), metabotropic glutamate receptor subtype 2 (8), and heparan sulphate (9). This determines the neurotropism of RABV and stimulates the body to produce cellular and humoral immune responses, which makes RABV-G an important antigen in the development of rabies vaccines (10, 11).

In the 1980s, an inactivated rabies vaccine was developed from the Flury Low Egg Passage (LEP) strain of RABV cultured in chicken embryos. The vaccine showed acceptable immunogenicity and tolerability and has been widely used ever since (12, 13). However, the production of chicken embryo cell rabies vaccines is limited by the supply of specific-pathogen-free chicken eggs and the relatively long production cycle. Other types of rabies vaccines used in large-scale vaccination programs include vaccines prepared in cell cultures of primary hamster kidney cells (14), human diploid cells (15, 16), and Vero cells (17, 18). High RABV yields can be achieved in Vero cell cultures, and high rabies vaccine yields can be obtained through inactivation and purification processes (19). However, the vaccine products contain not only viral antigens but also residual proteins and DNA of the Vero host cells. The residual proteins may trigger allergic reactions in the human body, while the residual host cell DNA may retain transforming activity (20, 21). The rabies vaccine produced using human diploid cells has shown good tolerability and immunogenicity in both humans and animals, but its large-scale production is complex and associated with lower virus yields and higher production costs than when other cell systems are used (13). Currently marketed RABV vaccines are mostly inactivated rabies vaccines, which carry a risk of incomplete virus inactivation either due to errors in the production process or a lack of stringency in quality control. Therefore, novel rabies vaccines that provide key assurances for the prevention of rabies must be developed. Previous studies have constructed RABV-G-expressing recombinant viruses (e.g., poxviruses and baculoviruses) that can protect mice against live-virus challenge and have verified the protective effect of RABV-G (22–24). However, viral vector vaccines for rabies prevention have not yet been marketed.

Unlike coronavirus disease 2019 (COVID-19) vaccines, such inactivated vaccines, mRNA vaccines, and recombinant subunit vaccines, which are produced by multiple technological approaches, there are currently no marketed recombinant subunit vaccines for rabies prevention. Researchers have attempted to produce RABV-G using prokaryotic or eukaryotic expression systems. Although the produced RABV-G exhibited reactogenicity by binding to specific antibodies, it failed to elicit high-level immune responses due to poor immunogenicity (25, 26). This may be related to the difficulty of performing correct post-translational modifications (e.g., protein glycosylation and folding) of RABV-G expressed *in vitro* (26, 27). mRNA vaccines are a new type of vaccines composed of mRNA that encodes the target antigen and a delivery system with an encapsulation-release function. They have shown outstanding protective effects against COVID-19 and have been widely used for protection against this disease (28). Preventive mRNA vaccines targeting respiratory syncytial virus (29, 30) and influenza virus (31) and mRNA vaccines for the treatment of cancers, such as melanoma and non-small cell lung cancer (32), are currently in clinical trials and have demonstrated the broad application prospects of mRNA vaccines. Unlike the production method for recombinant subunit vaccines, which involves the expression of recombinant proteins in either cells or bacteria cultured in bioreactors, mRNA vaccines enter somatic cells through endocytosis and release mRNA, which subsequently expresses the target antigen in the cells (33). Importantly, somatic cells provide a natural environment for post-translational modifications of proteins and the target antigen expressed *in vivo* may have stronger immunogenicity. This is crucial for glycosylation-dependent RABV-G-induced production of protective neutralizing antibodies. The traditional inactivated vaccine production process requires large-scale culture of live viruses and virus inactivation, which poses risks to workers and the production environment. In contrast, the mRNA vaccine production process does not involve cell or virus culture. Therefore, the production cycle is shorter and more suitable for large-scale vaccine production. Furthermore, mRNA vaccines also possess inherent adjuvant effects, eliminating the need for additional adjuvants to be included in the vaccine (33, 34). Non-replicating mRNA vaccines expressing RABV-G have been developed in recent years and have demonstrated good protective effects and tolerability in animal and clinical studies, confirming the feasibility of using mRNA vaccines for rabies prevention (35–37).

The CTN-1 strain of RABV was isolated in Shandong Province, China, in 1956. Its complete genome sequence shares 81.5%~93.4% sequence identity with vaccine and street strains worldwide. Specifically, the sequence similarity of the G protein of CTN-1 with that of other strains in China can be as high as 87.6%~97.7% (38). In the present study, we developed a non-replicating mRNA vaccine expressing the G protein of the CTN-1 strain of RABV. We monitored the levels of neutralizing antibodies induced and evaluated the protective effects of the vaccine against the live virus in mice, to provide a theoretical basis and supporting data for the development of mRNA-based rabies vaccines.

## 2 Materials and methods

### 2.1 Cells and viruses

BSR cells were purchased from the ATCC and stored at the National Institute for Food and Drug Control (NIFDC) in China. The cells were cultured in Dulbecco’s modified Eagle’s medium (DMEM, Gibco) containing 10% fetal bovine serum at 37°C in a 5% CO_2_ atmosphere. The standard RABV challenge strain CVS used for the lot release testing of rabies vaccines and the standard virus strain CVS-11 used for neutralizing antibody detection were both provided by the NIFDC.

### 2.2 Vaccines

**Figure 1:**
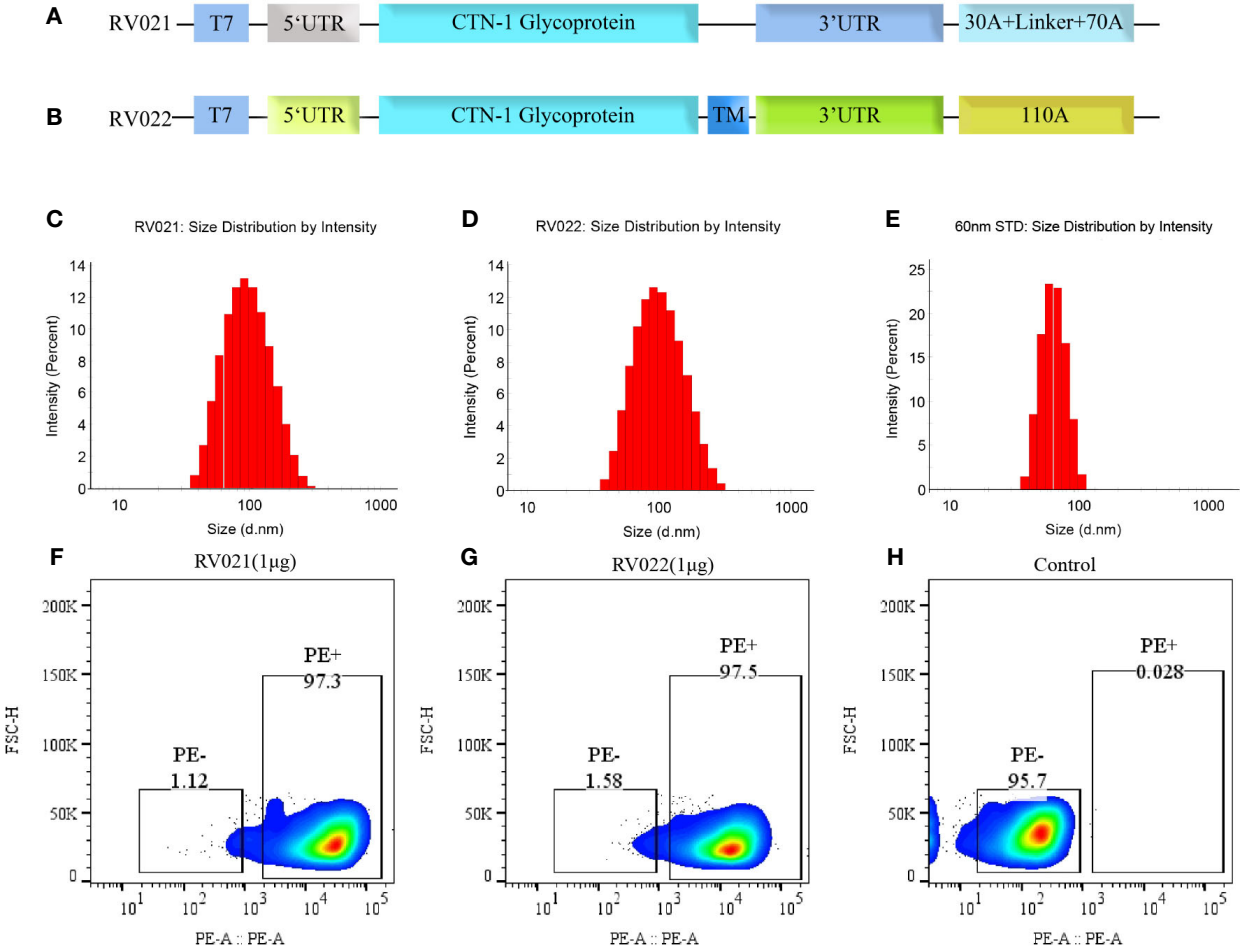
Antigen design, particle size, and *in vitro* expression of mRNA vaccines. **(A)** RV021 encodes the G protein of the CTN-1 strain, a monomeric glycoprotein containing non-coding sequences at both ends and a discontinuous poly **(A)** tail; **(B)** RV022 encodes the trimeric conformation of RABV-G with a different non-coding UTR design and a continuous 110A poly **(A)** tail. **(C-E)** Particle size distributions of RV021 **(C)**, RV022 **(D)**, and a 60-nm **(E)** standard measured by dynamic light scattering. **(F-H)** RABV-G expression in HEK293 cells transfected with RV021 **(F)**, RV022 **(G)**, or untransfected cells **(H)** as the negative control. T7: T7 promoter; UTR: untranslated region; and TM: trimerization motif.

mRNA sequences were optimized such that RV021 encoded the G protein (GenBank: JN234418.1) of the CTN-1 strain, and a trimerization motif was linked using a linker to the 3′ end of the G protein sequence of the RV022 coding region to express a G protein (GenBank: JN234418.1) with a trimeric conformation, and the trimerization sequence GYIPEAPRDGQAYVRKDGEWVLLSTFL was added to the C-terminus of the G protein. Both ends of the coding regions of the mRNA sequences contained untranslated regions (UTRs) and a poly(A) tail to enhance the mRNA translation efficiency (**Figures 1A, B**) (39). The sequences of RV021 and RV022, including the UTR and poly A tail, were synthesized by Genscript (Nanjing, China) and cloned into the pUC57 vector, resulting in the formation of two plasmids, pRV021 and pRV022, respectively. pRV021 and pRV022 were used as the transcription templates for mRNA after being digested with the restriction endonuclease Sap I. mRNA was synthesized using an *in vitro* transcription kit containing N1-Me-Pseudo-UTP (Cat# ON-040; Hongene Biotech, Shanghai, China) and a linear DNA plasmid as a template. A cap analog was added to the reaction system to add a cap-1 structure to the mRNA, and DNase I was added to remove the DNA template and terminate the reaction. The mRNA used in the *in vitro* transcription reaction system was purified using magnetic beads (Cat# N412-02, Vazyme, Nanjing, China), eluted in nuclease-free water, and then stored frozen. After purification, the mRNA was encapsulated with lipid nanoparticles (LNPs) to form an mRNA-LNP complex. The LNP mix was prepared by mixing the cationic lipids, 1,2-distearoyl-sn-glycero-3-phosphocholine (Sinopeg, Xiamen, China), cholesterol (AVT, Shanghai, China), and polyethylene glycol 2000 (Sinopeg, Xiamen, China) at a molar ratio of 50:10:38.5:1.5 and dissolving the mixture in anhydrous ethanol. The mRNA-LNP complex was prepared by rapidly mixing the purified mRNA with the LNP mix at a volume ratio of 3:1.

The inactivated rabies vaccine (IRV) used in this study was a freeze-dried Vero cell culture-derived IRV for human use with a potency of ≥2.5 IU/dose produced by Chengda Biotechnology Company (Liaoning, China). The 9th Chinese National Standard for Human Rabies Vaccine Potency (batch number: 250009-201909, potency: 11.4 IU/mL, 50% effective dose (ED_50_) range: 2.10–2.75), containing inactivated rabies virus and stabilizers, was used as the reference standard used for potency testing. LVRNA001 is an mRNA vaccine encoding the rabies virus G protein produced by Liverna Therapeutics Inc.(Zhuhai, China) (35).

### 2.3 Animal experiments

All animal experiments were approved by the Laboratory Animal Welfare and Ethics Committee of the NIFDC and performed in accordance with the guidelines provided by the committee. Specific-pathogen-free BALB/c mice aged 6~8 weeks and 20-day-old Kunming mice weighing 12~14 g were provided and reared by the NIFDC. RV021 and RV022 were administered to mice via intramuscular injections, with the injection volume fixed at 50 μL/mice and the dose adjusted by changing the vaccine concentration. Mice were vaccinated by intramuscular injection of the IRV at one-tenth of the human dose to measure humoral and cellular immune responses.

Vaccine potency was measured using the NIH test method. The IRV and potency reference standards were serially 5-fold diluted (e.g., 25-fold, 125-fold, and 625-fold) as according to the requirements of the Chinese Pharmacopoeia (40). Kunming mice were vaccinated via two intraperitoneal injections administered 7 days apart. The mRNA vaccines were also serially 5-fold diluted and subsequently used for the vaccination of Kunming mice via two intramuscular injections administered 7 days apart. On the 14th day after the booster vaccination, the mice were administered with 0.03 mL of live CVS virus via intracranial injection at a viral dose of 30~60-fold 50% lethal dose (LD_50_). The relative potency of the vaccines was determined by calculating the ED_50_ values of the test samples and reference standard.

### 2.4 Measurement of RABV-G expression by flow cytometry

Each LNP-encapsulated mRNA was transfected into 2 × 10^5^ HEK293T cells in a 24-well plate at 1 μg/well, and the cells were incubated at 37°C in a 5% CO_2_ atmosphere for 24 h. Following this, the cells were collected, fixed, and permeated using a fixation and permeabilization solution (Cat# 51-2090KZ; BD Biosciences, San Jose, USA), incubated with anti-RABV G antibody (1:500 dilution; Cat# PA5-117507; Invitrogen, Carlsbad, USA) at 4°C for 1 h to allow binding of the anti-RABV G antibody to the G protein expressed by the mRNA vaccine in the cells, and finally incubated with rabbit anti-mice immunoglobulin (Ig)G phycoerythrin (PE)-conjugated secondary antibody (1:500 dilution; Cat# P-2771MP; Invitrogen) at 4°C for 1 h. RABV-G expression was determined by flow-cytometric analysis of PE-positive cells.

### 2.5 Particle size measurement

LNP-encapsulated mRNA vaccines were diluted with phosphate-buffered saline (PBS, pH7.4; Cat#: P1020; Solarbio, Beijing, China), and placed in a cuvette for measurement of the particle size and particle dispersion coefficient by dynamic light scattering using a Malvern Zetasizer Nano-ZS. Scattered light was detected at a backscattering angle of 173°. Data were analyzed with the Zetasizer software.

### 2.6 Rapid fluorescent focus inhibition Test for serum neutralizing antibody titer detection against rabies virus

Rapid fluorescent focus inhibition test (RFFIT) and fluorescent antibody virus neutralization test (FAVNT) are recommended by the World Health Organization (WHO) for detecting neutralizing antibodies against RABV (41). In this study, RFFIT was used to determine the neutralizing antibody titer against rabies virus in mice serum according to Chinese Pharmacopoeia, as previously described (42). We use the sixth national standard of human immunoglobulin, prepared by NIFDC, as the biological reference standard, with the titer of 37 IU/mL. Mice serum and national standard were serially three-fold diluted in DMEM and added to a 96-well plate at 100 μL/well. The CVS-11 strain was diluted to a titer at which the fluorescence area reached 80–95% and was added to the diluted serum at 50 μL/well. The plate was incubated at 37°C for 1 h. BSR cells were added to the 96-well plate at a density of 5 × 10^5^ cells/well and cultured at 37°C in the presence of 5% CO_2_ for 24 h. After the supernatant was discarded, the cells were washed once with PBS and permeabilized using 80% cold acetone. The acetone was then discarded and a FITC-conjugated anti-RABV antibody (1:150 dilution; Cat# 5500; Millipore, Darmstadt, Germany) was added to the 96-well plate, which was then incubated at 37°C for 30 min. After washing with PBS, the fluorescence area was observed using a S6 Universal Fluorospot Analyzer (CTL, Cleveland, USA). The percentage of virus infection foci before and after 50% of cells infected was measured, and these were used to calculate the titer of the serum samples.

### 2.7 Specific IgG antibody titer measurement

The indirect enzyme-linked immunosorbent assay (ELISA) was used to measure the specific IgG antibody titer against rabies virus in the serum. The IRV was diluted 20-fold, coated onto a 96-well plate at 100 μL/well, and then incubated overnight. After the plate was washed with PBST (PBS + 0.05% Tween-20), the wells were blocked with PBST containing 1% bovine serum albumin at 37°C for 1 h. The plate was washed with PBST thrice, and three-fold diluted serum was added to the wells. After the plate was washed thrice with PBST, horseradish peroxidase-conjugated goat anti-mice IgG antibody (diluted 1:10,000) was added, and the plate was incubated at 37°C for 1 h. Finally, the plate was washed with PBST thrice, and 100 μL 3,3′,5,5′-tetramethylbenzidine (Cat# T5525; Sigma, Darmstadt, Germany) was added. 10 min later, 50 μL 2M H_2_SO_4_ was added to stop the reaction. The absorbance at 450 nm and 630 nm was measured using microplate reader (M200, TECAN, Männedorf, Switzerland). The serum IgG antibody titer was determined as the highest dilution at which the absorbance value was 2.1 times than that of the negative control and was calculated using GraphPad Prism v9.

### 2.8 Measurement of the cellular immune response by the enzyme-linked immunospot assay

Mice were dissected and their spleens were removed. Their spleens were then ground, treated with a lymphocyte separation solution (Cat# 7211011; DAKEWE, Shenzhen, China), and filtered through a 40-μm filter mesh. After the addition of RPMI-1640 culture medium (Cat# SH30096.01; Hyclone, Logan, USA), the mixture was centrifuged at 800× *g* for 30 min. Lymphocytes suspended in the middle layer were collected and stored in serum-free medium (Cat# 6015012; DAKEWE). The lymphocytes were counted using a Nexcelom Cellaca cell counter. The lymphocytes were added to a pre-coated ELISpot 96-well plate (Cat# 3321-4HPT-2; Mabtech, Nacka, Sweden) at a density of 2.5 × 10^5^ cells/well and were stimulated with a synthesized overlapped RABV-G peptide library (Genscript, Nanjing, China) for 24 h at 37°C. The results were analyzed using an ELISpot analyzer (S6 Universal, CTL, Cleveland, USA).

### 2.9 Statistical analysis

Graph plotting and statistical analysis were performed using GraphPad Prism v9. Data are expressed as geometric mean ± geometric standard deviation. Statistical differences among groups were determined by the one-way analysis of variance. Differences with *p* < 0.05 were considered statistically significant.

## 3 Results

### 3.1 mRNA vaccine design and construction

RABV-G is a key antigen that induces the production of neutralizing antibodies in the body. The G protein of the CTN-1 strain was used as the antigen and cloned into plasmids containing different UTRs and poly(A) tails after human codon optimization. Two plasmid templates for producing the candidate mRNA vaccines, pRV021 and pRV022, were prepared. Plasmid pRV021 has a poly A tail that was consistent with the expected length, while plasmid pRV022 has a poly A tail of 103 adenosines, which was slightly shorter than designed (**Supplementary Figure S1**). RV021 and RV022 were obtained through *in vitro* transcription. The stability and strong expression of the mRNA depended on the delivery system. Encapsulation of the mRNA stock with LNPs assists mRNA entry into the host cells and expression of the target antigen. The mean particle diameters of RV021 and RV022 were 89.0 nm and 86.8 nm, respectively, and their particle dispersion index values were 0.159 and 0.143, respectively. Micron-sized particles were not detected (**Figures 1C, D**). The mean particle diameters of the 60nm size standard samples meet the quality control requirements (**Figure 1E**), confirming the validity of the measurement results. RV021 and RV022 were directly transfected into HEK293T cells in 24-well plates at 1 μg/well. Untransfected cells were set as a negative control. Flow cytometry results showed that both mRNA vaccines were highly expressed *in vitro*, with positive cell rates of 97.3% and 97.5%, respectively (**Figures 1F, G**). In contrast, non-transfected cells exhibited only a 0.28% positivity rate (**Figure 1H**).

### 3.2 Candidate mRNA vaccines elicit humoral immune responses in mice

**Figure 2:**
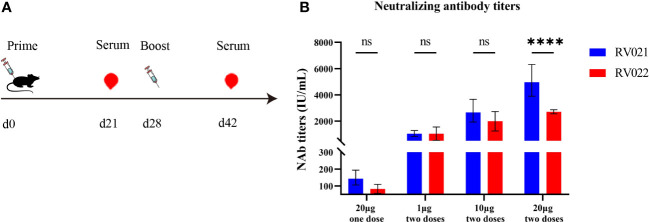
Measurement of neutralizing antibody titer against RABV by RFFIT. **(A)** Vaccination and blood sampling protocol. Mice were administered with two doses of mRNA vaccine at different dose levels 28 days apart, and blood samples were collected on days 21 and 42, respectively. **(B)** One-dose or two-dose (28 days apart) RV021 and RV022 induced neutralizing antibody titer in 6-week-old BALB/c mice (*n* = 5). **** *p* < 0.0001, ns: no significant difference.

Six-to-eight-week-old female BALB/c mice were randomly divided into seven groups and vaccinated on days 0 and 28 with the candidate mRNA vaccines RV021 and RV022 via intramuscular injection at doses of 1 μg, 10 μg, and 20 μg, respectively. On day 14 after the booster vaccination, blood samples were collected and sera were then separated for the evaluation of humoral immune responses. In the 20 μg group, additional blood samples were collected once on day 21 after the first vaccination. The neutralizing antibody titers in the sera were evaluated by RFFIT, and serum IgG antibody titers were evaluated by indirect ELISA. The results showed that both candidate mRNA vaccines elicited strong humoral immune responses and exhibited clear dose-dependent effects. The geometric mean titers (GMTs) of the serum neutralizing antibodies in the 20 μg one-dose, 1 μg two-dose, 10 μg two-dose, and 20 μg two-dose groups that received the RV021 vaccine were 143.3 IU/mL, 1,044 IU/mL, 2,666 IU/mL, and 4,968 IU/mL, respectively (**Figure 2**). The GMTSs of serum neutralizing antibodies in the 20 μg one-dose, 1 μg two-dose, 10 μg two-dose, and 20 μg two-dose groups that received the RV022 vaccine were 79.83 IU/mL, 991.7 IU/mL, 1,905 IU/mL, and 2,713 IU/mL, respectively. The highest GMTS of serum neutralizing antibodies in the 20 μg RV021 two-dose group was 4,968 IU/mL, which was found to be significantly higher than the titer of 2,713 IU/mL in the 20 μg RV022 two-dose group (*p* < 0.0001). Therefore, RV021 was utilized in subsequent experiments.

**Figure 3:**
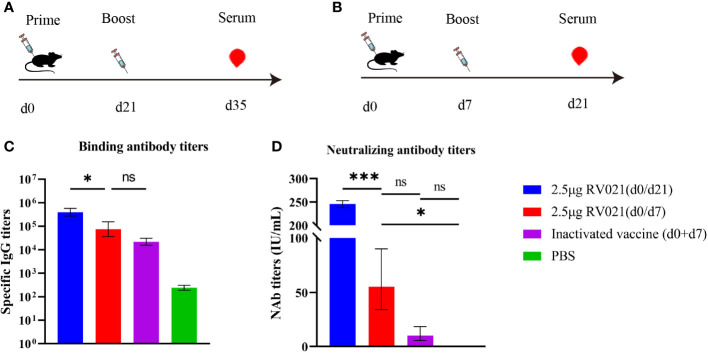
Humoral immune responses elicited by rabies mRNA vaccine and IRV. **(A)** Vaccination and blood sampling protocol. Six-week-old BALB/c mice were vaccinated with two doses of a rabies vaccine 21 days apart, and blood samples were collected on day 35 after the first vaccination (*n* = 5). **(B)** Vaccination and blood sampling protocol. Six-week-old BALB/c mice were vaccinated with two doses of a rabies vaccine 7 days apart, and blood samples were collected on day 21 after the first vaccination (*n* = 5). **(C)** IgG levels in mice as measured by ELISA. **(D)** Serum neutralizing antibody levels in mice as measured by RFFIT. * *p* < 0.05, *** *p* < 0.001, ns: no significant difference.

We designed another vaccination protocol involving the administration of two doses of RV021 with a 7-day or 21-day interval, and two doses of IRV with a 7-day interval. Mice serum was collected at 14 days after the booster vaccination to evaluate the humoral immune responses. The GMTs of serum neutralizing antibodies induced by two doses of 2.5 μg RV021 administered with a 7-day or 21-day interval, were 55.26 IU/mL and 245.6 IU/mL, respectively, and the serum IgG binding antibody titers were 74,634 and 395,797, respectively (**Figure 3**). In mice that received two doses of IRV with a 7-day interval, the GMTS and IgG antibody titers were 10.13 IU/mL and 21,798, respectively (**Figure 3**), both of which were lower than those in the mRNA vaccine group. This suggests that the mRNA vaccine had better immunogenicity than the IRV. The levels of IgG and neutralizing antibodies induced by two doses of 2.5 μg RV021 administered with a 21-day interval were found to be significantly higher than those induced with a 7-day interval.

**Figure 4:**
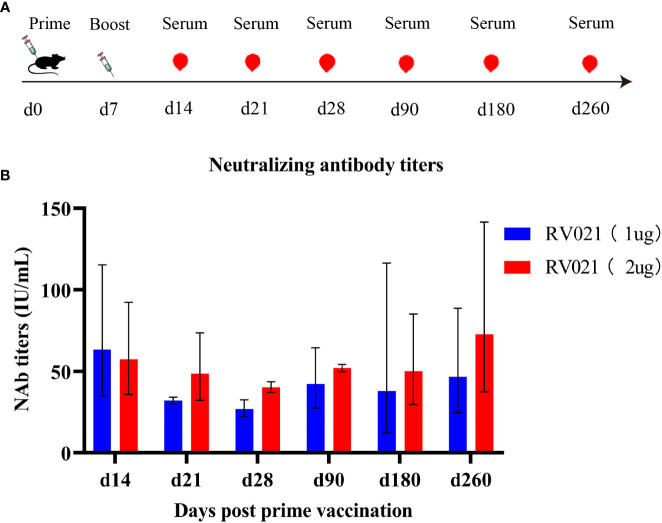
Persistence of neutralizing antibodies against RABV induced in mice by RV021. **(A)** Vaccination and blood sampling protocol. Six-week-old BALB/c mice were vaccinated with two doses of RV021 seven days apart, and blood samples were collected on days 14, 21, 28, 90, 180, and 260 after the first vaccination, respectively (*n* = 3 or 4). **(B)** Neutralizing antibody titers against RABV as measured by RFFIT.

We next investigated the persistence of neutralizing antibodies induced by the mRNA vaccine. Both 1 μg and 2 μg of RV021 induced high levels of neutralizing antibodies in mice on day 7 after the booster vaccination, with GMTSs of 71 IU/mL and 61 IU/mL, respectively (**Figure 4**). Antibody levels were monitored until day 260 after the booster vaccination, when the neutralizing antibody titers for the 1 μg and 2 μg groups were 46.7 IU/mL and 72.8 IU/mL, respectively. These levels were found to be substantially higher than the WHO-accepted threshold titer of 0.5 IU/mL, indicating that the protective effect of RV021 at doses of 1 μg or 2 μg can be maintained for at least 260 days, with no observed trend of decrease in the antibody titer.

### 3.3 RV021 Elicits a strong cellular immune response in mice

**Figure 5:**
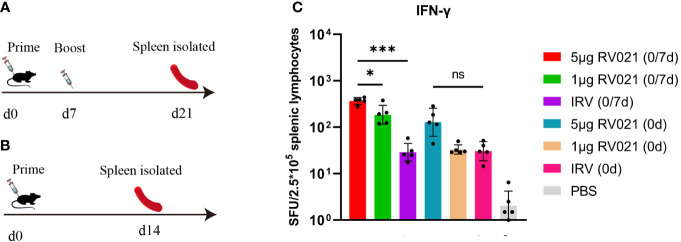
Cellular immune response elicited by RV021 and IRV in mice. **(A, B)** Vaccination and experimental protocol. Mice received either two doses 7 days apart **(A)** or only one dose **(B)** of the rabies vaccine. On day 14 after the completion of vaccination, the mice were sacrificed, and their spleens were removed (*n* = 5). **(C)** Levels of secreted IFN-γ in mice lymphocytes as measured by the ELISpot assay. * *p* < 0.05, *** *p* < 0.001, ns: no significant difference. SFU, spot-forming unit.

Six-week-old female BALB/c mice were vaccinated with either two doses of RV021 or IRV at a 7-day interval, or only one dose of RV021 or IRV. On day 14 after the completion of the vaccination protocol, the spleens of the mice were removed to isolate the lymphocytes, and IFN-γ secretion was measured using the ELISpot assay (**Figures 5A, B**). Vaccination with 5 μg of RV021 seven days apart induced the highest level of cellular immunity, with a geometric mean of 368 spot-forming units (SFUs) per 2.5 × 10^5^ lymphocytes (**Figure 5C**). This was significantly higher than that of two-dose IRV (SFUs = 29, *p* = 0.0007). The cellular immunity level in the one-dose 5 μg RV021 group was 128 SFUs, which was higher than that of the one-dose IRV group (31 SFUs), albeit not significantly (*p* > 0.05). Cellular immunity in the 1 μg RV021 (0/7 d) group was 186 SFUs, which was higher than that in the one-dose 5 μg RV021 (0 d) group. These results indicated that the rabies mRNA vaccine RV021 at 5 μg has the potential to elicit stronger cellular immune responses than IRVs. A change from a single dose to two doses of RV021 caused a more significant improvement in the cellular immune response than an increase in single dose from 1 μg to 5 μg.

### 3.4 Low-Dose RV021 protects mice against live-virus challenge

In a preliminary investigation of the effects of mRNA vaccines on live-virus challenge, 6-week-old BALB/c mice were randomly divided into seven groups. On days 0 and 28, the mice were administered intramuscular injections of 1 μg, 10 μg, or 20 μg of the mRNA vaccines RV021 and RV022, respectively. Twenty-eight days after the booster vaccination, the mice were challenged with live RABV via intracranial injection of CVS at 30~60-fold LD_50_ and were observed for 14 days. All five mice in the control group (Empty-LNP) died within 14 days after the virus challenge. In contrast, all mice in the remaining six groups, including the two groups vaccinated with only 1 μg of vaccine, survived without showing any signs of disease onset (**Table 1**).

**Table 1:** Protective effects of RV021 and RV022 against live-virus challenge.

Administration	RV021			RV022			Empty-LNP
Dose	1 μg	10 μg	20 μg	1 μg	10 μg	20 μg	0
Live/Total	5/5	5/5	5/5	5/5	5/5	5/5	0/5

The vaccination strategy of two doses of mRNA vaccine on days 0 and 28 elicited a strong humoral immune response and protected against virus challenge. However, pre-exposure prophylaxis against human RABV infection often involves booster vaccination on day 7. Two doses of the vaccine were administered to 20-day-old Kunming mice weighing 12–14 g via intramuscular injection 7 days apart. Seven days after the boost vaccination, the mice received an intracranial injection of the standard challenge virus CVS at 30~60-fold LD_50_. Another mRNA vaccine presently under development (LVRNA001) (35) and the national standard (as a control) were used to investigate the protective effect of the mRNA vaccines against virus challenge. All mice in the PBS control group died within 14 days after the virus challenge, whereas all mice vaccinated with the mRNA vaccines RV021 and LVRNA001 survived, regardless of the dose (1 μg or 2 μg) (**Table 2**). The log ED_50_ of the protective effect produced by the national standard was 2.63, which is within the calibration range. This demonstrates the validity of the experimental results, i.e., intramuscular injection of two doses of 1 μg RV021 seven days apart provided 100% protection.

**Table 2:** Protective effects of the mRNA vaccines RV021 and LVRNA001 against live-virus challenge.

Group	Administration	Dose	Live/Total	Survival Rate
1	RV021	1 μg	16/16	100%
2		2 μg	16/16	100%
3	LVRNA001	1 μg	16/16	100%
4		2 μg	16/16	100%
5	9th Std ^1^	1/25	15/16	93.8%
6		1/125	16/16	100%
7		1/625	6/16	37.5%
8	PBS	0	0/16	0%

^1^ 9th Chinese National Standard for human rabies vaccine potency.

The candidate mRNA vaccine series was further diluted to explore the protective effects of lower doses of the vaccine against virus challenge. Kunming mice aged 20 days and weighing 12–14 g were randomly divided into groups of 16 mice each. Vaccination was performed on days 0 and 7 with different doses of RV021. Seven days after the booster vaccination, the mice received intracranial injections of the standard challenge virus CVS at 30~60-fold LD_50_ and were observed for another 14 days. At extremely low vaccine doses of 0.008–0.8 μg, the survival rate ranged from 12.5%–100% (**Table 3**). Four-parameter fitting of the survival results after the virus challenge revealed a significant correlation between the survival rate and vaccination dose, with R^2 =^ 0.93273. The ED_50_ of RV021 was 0.031 μg (**Supplementary Figure S2**), indicating that RV021 provides a good protective effect even at extremely low doses.

**Table 3:** Protective effects of the low-dose series of RV021 against live-virus challenge.

Group	Dose	Live/Total	Survival Rate
1	0.8 μg	16/16	100%
2	0.4 μg	16/16	100%
3	0.2 μg	15/16	93.75%
4	0.16 μg	16/16	100%
5	0.08 μg	11/16	68.75%
6	0.04 μg	7/16	43.75%
7	0.032 μg	9/16	56.25%
8	0.016 μg	7/16	43.75%
9	0.008 μg	2/16	12.5%
10	PBS	0/16	0%

### 3.5 RV021 at relatively low human doses has a potency that meets the standards for human vaccine release

The potency of RV021 at different human doses (5, 10, 15, and 50 μg/dose, respectively) was measured using the potency determination method for human rabies vaccines (NIH method), as specified in the 2020 edition of the Chinese Pharmacopoeia. Our previous study showed that the potency of RV021 at a human dose of 50 μg determined using the NIH method was >16.1 IU/dose, whereas that of the IRV was >13.6 IU/dose (**Supplementary Table S1**). In the present study, we further determined the potency of RV021 at low doses. The potency of RV021 in human doses of 5 μg/dose and 10 μg/dose was 2.1 IU/dose and 3.9 IU/dose, respectively (**Table 4**). According to the Chinese Pharmacopoeia, the required potency for human rabies vaccines during shelf-life and at release is ≥2.5 IU/dose and ≥4.0 IU/dose, respectively. The potency of RV021 at 15 μg/dose was 7.5 IU/dose, which meets the standard for lot release.

**Table 4:** Potency of RV021 as measured using the NIH method.

Group	Dilution	Survival		Accumulated			Result	
		Death	Live	Death	Live	Death Rate	Log ED_50_	Potency
RV021 (5 μg/dose)	×25	4	12	4	23	14.8%	2.09	2.1 IU/dose
	×125	5	11	9	11	45%		
	×625	16	0	25	0	100%		
RV021 (10 μg/dose)	×25	2	14	2	29	6.5%	2.36	3.9 IU/dose
	×125	2	14	4	15	21.1%		
	×625	15	1	19	1	95%		
RV021 (15 μg/dose)	×25	0	16	0	38	0%	2.64	7.5 IU/dose
	×125	0	16	0	22	0%		
	×625	10	6	10	6	62.5%		
9th Std ^1^	×25	1	15	1	34	2.9%	2.52	11.4 IU/mL
	×125	3	13	4	19	17.4%		
	×625	10	6	14	6	70%		
PBS	\	16	0	\	\	100%	\	\

^1^ 9th Chinese National Standard for human Rabies vaccines potency.

## 4 Discussion

In recent years, immense progress has been achieved in the improvement of the production capacity and quality control of human rabies vaccines. However, research efforts have mainly been focused on inactivated vaccines. mRNA vaccine production differs from traditional vaccines as it does not involve the cultivation of cells or viruses. Consequently, it boasts a shorter production cycle, robust process scalability, and is well-suited for industrialization (28, 33). In the present study, we formulated two RABV-G-expressing rabies mRNA vaccines, RV021 and RV022. The results of *in vivo* mice experiments revealed that both mRNA vaccines have a good immunogenicity and induce strong humoral and cellular immune responses in mice.

We first compared the immunogenicity induced by the candidate vaccines RV021 and RV022 and found that RV021 induced higher neutralizing antibody levels than RV022 in mice. RABV-G forms approximately 400 trimeric spikes tightly arranged on the virus surface that play a role in the virus binding to host cell receptors (43). To mimic the natural trimeric conformation of RABV-G, we introduced a trimerization motif at the 3′ end of the coding region of RV022. This trimerization approach has previously been successfully applied in research on mRNA vaccines for COVID-19 (44). The reduced immunogenicity of the trimeric G protein compared to that of the monomeric form may have caused the neutralizing antibody level induced by RV022 to be significantly lower than that induced by RV021. Several studies have reported good immunogenicity with the use of the monomeric G protein in rabies mRNA vaccines (35, 37, 45). However, the antigen-coding and non-coding regions of RV021 and RV022 were designed differently, making it difficult to accurately pinpoint the cause of their difference in immunogenicity. RV022 possesses a poly(A) tail containing 110 consecutive adenosines, but sequencing results indicated that the plasmid pRV022 had 103 adenosines, which may be due to recombination during bacterial amplification of the plasmid DNA, as well as the inaccuracies in determining the length of repetitive sequences during Sanger sequencing. The difference between the 103 adenosines and 110 adenosines may also not be the main reason for the lower RV022 neutralizing antibody levels observed compared to RV021. The poly(A) structure of mRNA vaccines is designed in the plasmid in the upstream process. Owing to the instability of long repetitive sequences at the ends, truncation of the poly(A) tail often occurs during plasmid fermentation. Studies have revealed that segmented poly(A) tails containing spacers can improve the stability of plasmid DNA and the *in vivo* expression efficiency of mRNA (46–48). Therefore, a segmented poly(A) tail design was adopted in the candidate vaccine RV021, which enabled the retention of an intact poly(A) tail in the plasmid preparation. The design of non-coding sequences at the 5′ and 3′ ends may also affect the immunogenicity of mRNA vaccines. Factors that affect immunogenicity can therefore be investigated by designing mRNA vaccines with various types of sequences.

For pre-exposure prophylaxis using IRV, booster vaccination was performed on day 7 after the first vaccination, and the protective antibody titer effective against RABV should be ≥0.5 IU/mL (45). In this study, the antibody titers induced by RV021 at different vaccination intervals were found to be considerably higher than 0.5 IU/mL and were maintained for at least 260 days. Vaccination of mice with two doses at different intervals (7, 21, and 28 days) induced varying levels of neutralizing antibodies, with the levels increasing accordingly with prolongation of the interval. Similarly, Grunau et al. found that prolongation of the interval between two doses of mRNA vaccines for COVID-19 significantly increased the antibody levels of vaccinated individuals (49). In this study, we found that two doses of mRNA vaccine significantly improved antibody levels compared to a single dose, i.e., the vaccination strategy of two low doses was slightly superior to the single high-dose strategy. This result is of great significance as it indicates that the dose of mRNA vaccines can be lowered to further reduce toxicity and production costs.

The IRV production technology is relatively mature and has broad applications. However, studies have shown that rabies mRNA vaccines have superior immunogenicity over IRVs, particularly in their potential to induce cellular immune responses (35, 45). In this study, BALB/c mice were vaccinated with 2.5 μg of RV021 seven days apart. On day 14 after the booster vaccination, the GMTs of neutralizing antibodies in the mice was 55.26 IU/mL, whereas the GMTs of neutralizing antibodies in mice that received IRV using the same vaccination protocol was 10.13 IU/mL. This demonstrates that the mRNA vaccine has better immunogenicity than the IRV. RV021 also elicited cellular immune responses as manifested by high IFN-γ secretion levels, which were found to be significantly higher than those induced by the IRV and were consistent with results reported in the literature (35). However, the persistence of the cellular immune response induced by RV021 should be further studied.

Live-virus challenge experiments can effectively reflect the true protective effects of vaccines. In our virus challenge experiment in mice, RV021 exhibited good protective effects even at low doses. In mice vaccinated with two doses of RV021 seven days apart at different dose levels and subsequently intracranially challenged with live RABV at 30–60-fold LD_50_, the ED_50_ of RV021 was 0.031 μg. The NIH method, developed by the NIH in the USA, is a classic assay currently used for rabies vaccine potency evaluation. For rabies vaccines, a potency of ≥4.0 IU/dose is considered to be protective. At present, there are no approved rabies mRNA vaccines worldwide, and therefore, reference dose levels are lacking. The dose levels of the COVID-19 mRNA vaccines BNT162b2 and mRNA-1273, which have been widely used, are 30 μg/dose and 100 μg/dose, respectively (33). However, adverse reactions, such as myocarditis (50, 51), and skin reactions (52), may occur. The use of a low dose may be an important means to reduce the incidence of adverse reactions associated with mRNA vaccines. Data from live-virus challenge experiments in animals vaccinated with rabies mRNA vaccines have been reported, but there is a lack of research on the correlation between the relative potency and the mRNA dose. In the present study, the NIH method was, for the first time, used to determine the potency of rabies RNA vaccines, and it was found that the vaccine dose was highly correlated with the potency. At human doses of 10 μg/dose and 15 μg/dose, the potency of RV021 was 3.9 IU/dose and 7.5 IU/dose, respectively. This indicates that RV021 provides an acceptable protective effect at a dose of 15 μg/dose.

Further work expanding on the present study is required. RABV-N can activate B cell proliferation and induce T cell responses. However, in this study, we designed the mRNA vaccines encoding solely RABV-G as the antigen. Co-expression of RABV-N and RABV-G in the mRNA vaccine may potentially enhance cellular immunity, induce the long-lasting immune memory, and be more suitable for pre-exposure prevention of rabies. Post-exposure prophylaxis is a key application scenario for the administration of rabies vaccines. Therefore, the credibility of our results could be further enhanced through investigation of the protective effects of RV021 in post-exposure prophylaxis. Studies on the effectiveness and safety of vaccines in various animal models are also an integral part of rabies mRNA vaccine research. In conclusion, we successfully prepared a rabies mRNA vaccine that induces long-lasting neutralizing antibodies and provides effective protection even at low doses. Our results provide a novel approach and data for further development of rabies mRNA vaccines, which will contribute to the diversification of the rabies vaccine pipeline.

## Data availability statement

The original contributions presented in the study are included in the article/**Supplementary Materials**, further inquiries can be directed to the corresponding authors.

## Ethics statement

Ethical approval was not required for the studies on humans in accordance with the local legislation and institutional requirements because only commercially available established cell lines were used. The animal study was approved by the Laboratory Animal Welfare and Ethics Committee of the NIFDC. The study was conducted in accordance with the local legislation and institutional requirements.

## Author contributions

ML: Conceptualization, Visualization, Writing – original draft. EF: Conceptualization, Writing – original draft. YW: Conceptualization, Writing – original draft. LS: Conceptualization, Writing – original draft. JL: Conceptualization, Writing – original draft. QP: Methodology, Writing – original draft. XXL: Methodology, Writing – original draft. DZ: Software, Writing – original draft. XHL: Software, Writing – original draft. XYL: Software, Writing – original draft. JJL: Validation, Writing – original draft. HX: Validation, Writing – original draft. HW: Validation, Writing – original draft. YH: Formal Analysis, Writing – original draft. RY: Investigation, Writing – original draft. GY: Resources, Writing – original draft. YS: Data curation, Writing – original draft. XW: Writing – review & editing. SC: Supervision, Writing – review & editing. YL: Project administration, Writing – review & editing.

## References

[1] Hampson K Coudeville L Lembo T Sambo M Kieffer A Attlan M. Estimating the global burden of endemic canine rabies. PloS Negl Trop Dis (2015) 9(4):e0003709. doi: 10.1371/journal.pntd.0003709 25881058 PMC4400070

[2] Hemachudha T Ugolini G Wacharapluesadee S Sungkarat W Shuangshoti S Laothamatas J. Human rabies: neuropathogenesis, diagnosis, and management. Lancet Neurol (2013) 12(5):498–513. doi: 10.1016/s1474-4422(13)70038-3 23602163

[3] Raux H Flamand A Blondel D. Interaction of the rabies virus P protein with the LC8 dynein light chain. J Virol (2000) 74(21):10212–6. doi: 10.1128/jvi.74.21.10212-10216.2000 PMC102061 11024151

[4] Fooks AR Banyard AC Horton DL Johnson N McElhinney LM Jackson AC. Current status of rabies and prospects for elimination. Lancet (2014) 384(9951):1389–99. doi: 10.1016/s0140-6736(13)62707-5 PMC7159301 24828901

[5] Fisher CR Streicker DG Schnell MJ. The spread and evolution of rabies virus: conquering new frontiers. Nat Rev Microbiol (2018) 16(4):241–55. doi: 10.1038/nrmicro.2018.11 PMC6899062 29479072

[6] Thoulouze M-I Lafage M Schachner M Hartmann U Cremer H Lafon M. The neural cell adhesion molecule is a receptor for rabies virus. J Virol (1998) 72(9):7181–90. doi: 10.1128/JVI.72.9.7181-7190.1998 PMC109940 9696812

[7] Lentz TL Burrage TG Smith AL Crick J Tignor GH. Is the acetylcholine receptor a rabies virus receptor? Science (1982) 215(4529):182–4. doi: 10.1126/science.7053569 7053569

[8] Wang J Wang Z Liu R Shuai L Wang X Luo J. Metabotropic glutamate receptor subtype 2 is a cellular receptor for r abies virus. PloS Pathog (2018) 14(7):e1007189. doi: 10.1371/journal.ppat.1007189 30028877 PMC6070288

[9] Sasaki M Anindita PD Ito N Sugiyama M Carr M Fukuhara H. The role of heparan sulfate proteoglycans as an attachment factor for rabies virus entry and infection. J Infect Dis (2018) 217(11):1740–9. doi: 10.1093/infdis/jiy081 29529215

[10] Ng WM Fedosyuk S English S Augusto G Berg A Thorley L. Structure of trimeric pre-fusion rabies virus glycoprotein in complex with two protective antibodies. Cell Host Microbe (2022) 30(9):1219–30.e7. doi: 10.1016/j.chom.2022.07.014 35985336 PMC9605875

[11] Fodor E. Insight into the multifunctional RNA synthesis machine of rabies virus. Proc Natl Acad Sci U.S.A. (2020) 117(8):3895–7. doi: 10.1073/pnas.2000120117 PMC7049141 31992635

[12] Barth R Gruschkau H Bijok U Hilfenhaus J Hinz J Milcke L. A new inactivated tissue culture rabies vaccine for use in man. Evaluation of PCEC-vaccine by laboratory tests. J Biol Stand (1984) 12(1):29–46. doi: 10.1016/s0092-1157(84)80019-0 6199357

[13] Giesen A Gniel D Malerczyk C. 30 Years of rabies vaccination with Rabipur: a summary of clinical data and global experience. Expert Rev Vaccines (2015) 14(3):351–67. doi: 10.1586/14760584.2015.1011134 25683583

[14] Lin F Zeng F Lu L Lu X Zen R Yu Y. The primary hamster kidney cell rabies vaccine: adaptation of viral strain, production of vaccine, and pre- and postexposure treatment. J Infect Dis (1983) 147(3):467–73. doi: 10.1093/infdis/147.3.467 6833794

[15] Plotkin SA. Vaccine production in human diploid cell strains. Am J Epidemiol (1971) 94(4):303–6. doi: 10.1093/oxfordjournals.aje.a121323 4329326

[16] Wiktor TJ Koprowski H. Successful immunization of primates with rabies vaccine prepared in human diploid cell strain WI-38. Proc Soc Exp Biol Med (1965) 118:1069–73. doi: 10.3181/00379727-118-30048 14280692

[17] Fisher CR Schnell MJ. New developments in rabies vaccination. Rev Sci Tech (2018) 37(2):657–72. doi: 10.20506/rst.37.2.2831 30747119

[18] Moulenat T Petit C Bosch Castells V Houillon G. Purified vero cell rabies vaccine (PVRV, Verorab(^®^)): A systematic review of intradermal use between 1985 and 2019. Trop Med Infect Dis (2020) 5(1):40. doi: 10.3390/tropicalmed5010040 32156005 PMC7157209

[19] Barrett PN Mundt W Kistner O Howard MK. Vero cell platform in vaccine production: moving towards cell culture-based viral vaccines. Expert Rev Vaccines (2009) 8(5):607–18. doi: 10.1586/erv.09.19 19397417

[20] Organization WH. WHO expert committee on biological standardization: sixty-first resport. WHO Tech Rep Ser (2010) 978:131–7.

[21] Sheng-Fowler L Lewis AM Jr. Peden K. Issues associated with residual cell-substrate DNA in viral vaccines. Biologicals (2009) 37(3):190–5. doi: 10.1016/j.biologicals.2009.02.015 19285882

[22] Zhao C Gao J Wang Y Ji L Qin H Hu W. A novel rabies vaccine based on a recombinant bovine herpes virus type 1 expressing rabies virus glycoprotein. Front Microbiol (2022) 13:931043. doi: 10.3389/fmicb.2022.931043 35755997 PMC9213812

[23] Kieny MP Lathe R Drillien R Spehner D Skory S Schmitt D. Expression of rabies virus glycoprotein from a recombinant vaccinia virus. Nature (1984) 312(5990):163–6. doi: 10.1038/312163a0 6548799

[24] Wiktor TJ Macfarlan RI Reagan KJ Dietzschold B Curtis PJ Wunner WH. Protection from rabies by a vaccinia virus recombinant containing the rabies virus glycoprotein gene. Proc Natl Acad Sci USA. (1984) 81(22):7194–8. doi: 10.1073/pnas.81.22.7194 PMC392104 6095272

[25] Zhao R Shan Y Li M Lou Z Feng Y Huang L. Novel strategy for expression and characterization of rabies virus glycoprotein. Protein Expression Purification (2020) 168:105567. doi: 10.1016/j.pep.2019.105567 31904423

[26] Askri H Akrouti I Rourou S Kallel H. Production, purification, and characterization of recombinant rabies virus glycoprotein expressed in PichiaPink yeast. Biotechnol Rep (Amst) (2022) 35:e00736. doi: 10.1016/j.btre.2022.e00736 35646619 PMC9130087

[27] Koraka P Bosch BJ Cox M Chubet R Amerongen G Lövgren-Bengtsson K. A recombinant rabies vaccine expressing the trimeric form of the glycoprotein confers enhanced immunogenicity and protection in outbred mice. Vaccine (2014) 32(36):4644–50. doi: 10.1016/j.vaccine.2014.06.058 24962755

[28] Chaudhary N Weissman D Whitehead KA. mRNA vaccines for infectious diseases: principles, delivery and clinical translation. Nat Rev Drug Discovery (2021) 20(11):817–38. doi: 10.1038/s41573-021-00283-5 PMC8386155 34433919

[29] Espeseth AS Cejas PJ Citron MP Wang D DiStefano DJ Callahan C. Modified mRNA/lipid nanoparticle-based vaccines expressing respiratory syncytial virus F protein variants are immunogenic and protective in rodent models of RSV infection. NPJ Vaccines (2020) 5(1):16. doi: 10.1038/s41541-020-0163-z 32128257 PMC7021756

[30] Tiwari PM Vanover D Lindsay KE Bawage SS Kirschman JL Bhosle S. Engineered mRNA-expressed antibodies prevent respiratory syncytial virus infection. Nat Commun (2018) 9(1):3999. doi: 10.1038/s41467-018-06508-3 30275522 PMC6167369

[31] Arevalo CP Bolton MJ Le Sage V Ye N Furey C Muramatsu H. A multivalent nucleoside-modified mRNA vaccine against all known influenza virus subtypes. Sci (New York NY) (2022) 378(6622):899–904. doi: 10.1126/science.abm0271 PMC10790309 36423275

[32] Miao L Zhang Y Huang L. mRNA vaccine for cancer immunotherapy. Mol Cancer (2021) 20(1):41. doi: 10.1186/s12943-021-01335-5 33632261 PMC7905014

[33] Fang E Liu X Li M Zhang Z Song L Zhu B. Advances in COVID-19 mRNA vaccine development. Signal Transduct Target Ther (2022) 7(1):94. doi: 10.1038/s41392-022-00950-y 35322018 PMC8940982

[34] Verbeke R Lentacker I De Smedt SC Dewitte H. Three decades of messenger RNA vaccine development. Nano Today (2019) 28:100766. doi: 10.1016/j.nantod.2019.100766

[35] Li J Liu Q Liu J Wu X Lei Y Li S. An mRNA-based rabies vaccine induces strong protective immune responses in mice and dogs. Virol J (2022) 19(1):184. doi: 10.1186/s12985-022-01919-7 36371169 PMC9652961

[36] Aldrich C Leroux-Roels I Huang KB Bica MA Loeliger E Schoenborn-Kellenberger O. Proof-of-concept of a low-dose unmodified mRNA-based rabies vaccine formulated with lipid nanoparticles in human volunteers: a phase 1 trial. Vaccine (2021) 39(8):1310–8. doi: 10.1016/j.vaccine.2020.12.070 PMC7825876 33487468

[37] Stitz L Vogel A Schnee M Voss D Rauch S Mutzke T. A thermostable messenger RNA based vaccine against rabies. PloS Negl Trop Dis (2017) 11(12):e0006108. doi: 10.1371/journal.pntd.0006108 29216187 PMC5737050

[38] Shi LT Yu YX Liu JH Tang JR Wu XH Cao SC. Analysis of full-length gene sequence of a rabies vaccine strain CTN-1 for human use in China. Bing Du Xue Bao (2010) 26(3):195–201. doi: 10.13242/j.cnki.bingduxuebao.002084 20572340

[39] van der Velden AW Thomas AA. The role of the 5’ untranslated region of an mRNA in translation regulation during development. Int J Biochem Cell Biol (1999) 31(1):87–106. doi: 10.1016/s1357-2725(98)00134-4 10216946

[40] Commission CP. Pharmacopoeia of the people’s republic of China 2020 edition. Beijing, China: China Press of Traditional Chinese Medicine Vol. 3. (2020). pp. 578–9.

[41] Organization WH. Recommendations for inactivated rabies vaccine for human use produced in cell substrates and embryonated eggs. WHO Tech Rep Ser (2007) 941:83–132.

[42] Yu P Lv X Shen X Tang Q Liang G. Establishment and preliminary application of a rapid fluorescent focus inhibition test (RFFIT) for rabies virus. Virol Sin (2013) 28(4):223–7. doi: 10.1007/s12250-013-3321-x PMC8208402 23913179

[43] Schnell MJ McGettigan JP Wirblich C Papaneri A. The cell biology of rabies virus: using stealth to reach the brain. Nat Rev Microbiol (2010) 8(1):51–61. doi: 10.1038/nrmicro2260 19946287

[44] Liang Q Wang Y Zhang S Sun J Sun W Li J. RBD trimer mRNA vaccine elicits broad and protective immune responses against SARS-CoV-2 variants. iScience (2022) 25(4):104043. doi: 10.1016/j.isci.2022.104043 35291264 PMC8915453

[45] Schnee M Vogel AB Voss D Petsch B Baumhof P Kramps T. An mRNA vaccine encoding rabies virus glycoprotein induces protection against lethal infection in mice and correlates of protection in adult and newborn pigs. PloS Negl Trop Dis (2016) 10(6):e0004746. doi: 10.1371/journal.pntd.0004746 27336830 PMC4918980

[46] Trepotec Z Geiger J Plank C Aneja MK Rudolph C. Segmented poly(A) tails significantly reduce recombination of plasmid DNA without affecting mRNA translation efficiency or half-life. Rna (2019) 25(4):507–18. doi: 10.1261/rna.069286.118 PMC6426288 30647100

[47] Florian E Ugur SA Kuhn A Britta VM Diken M. Stabilization of poly(a) sequence encoding dna sequences patent US 2017/0166905. (2017).

[48] Stadler CR Bähr-Mahmud H Celik L Hebich B Roth AS Roth RP. Elimination of large tumors in mice by mRNA-encoded bispecific antibodies. Nat Med (2017) 23(7):815–7. doi: 10.1038/nm.4356 28604701

[49] Grunau B Goldfarb DM ASamoah-Boaheng M Golding L Kirkham TL Demers PA. Immunogenicity of extended mRNA SARS-CoV-2 vaccine dosing intervals. JAMA (2022) 327(3):279–81. doi: 10.1001/jama.2021.21921 PMC8642809 34860253

[50] Mevorach D Anis E Cedar N Bromberg M Haas EJ Nadir E. Myocarditis after BNT162b2 mRNA Vaccine against Covid-19 in Israel. New Engl J Med (2021) 385(23):2140–9. doi: 10.1056/NEJMoa2109730 PMC8531987 34614328

[51] Bozkurt B Kamat I Hotez PJ. Myocarditis with COVID-19 mRNA vaccines. Circulation (2021) 144(6):471–84. doi: 10.1161/CIRCULATIONAHA.121.056135 PMC8340726 34281357

[52] Pasternack R Pohjavaara S. A skin reaction with rust-like discolouration to mRNA COVID-19 vaccine. J Eur Acad Dermatol Venereol (2021) 35(11):e737–e8. doi: 10.1111/jdv.17543 PMC8447431 34310755

