# Supplementary material for: An mRNA vaccine against rabies provides strong and durable protection in mice
Source: Front Immunol. 2023 Oct 26;14:1288879. doi: 10.3389/fimmu.2023.1288879 (PMC10639119; doi:10.3389/fimmu.2023.1288879)
Supplement: Supplementary file 1 [file DataSheet_1.docx]

An mRNA Vaccine against Rabies That Provides Strong and Durable Protection in Mice


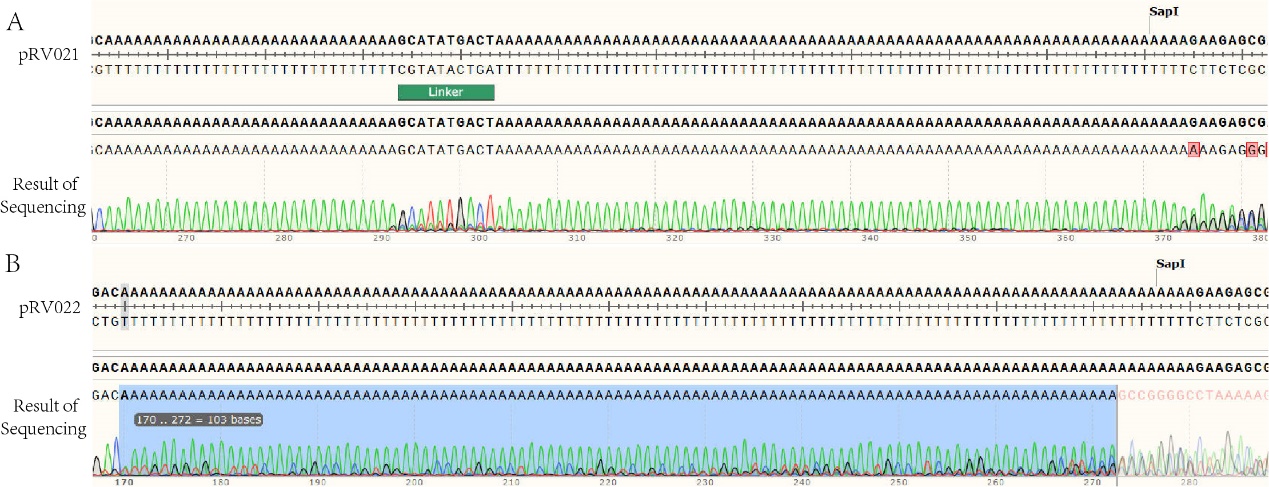


**Figure S1.** Alignment of poly(A) tail sequencing results with plasmid templates. **(A)** Poly(A) tail sequencing results of plasmid pRV021. **(B)** Poly(A) tail sequencing results of plasmid pRV022. Sequencing results were aligned using SnapGene 6.0.2.


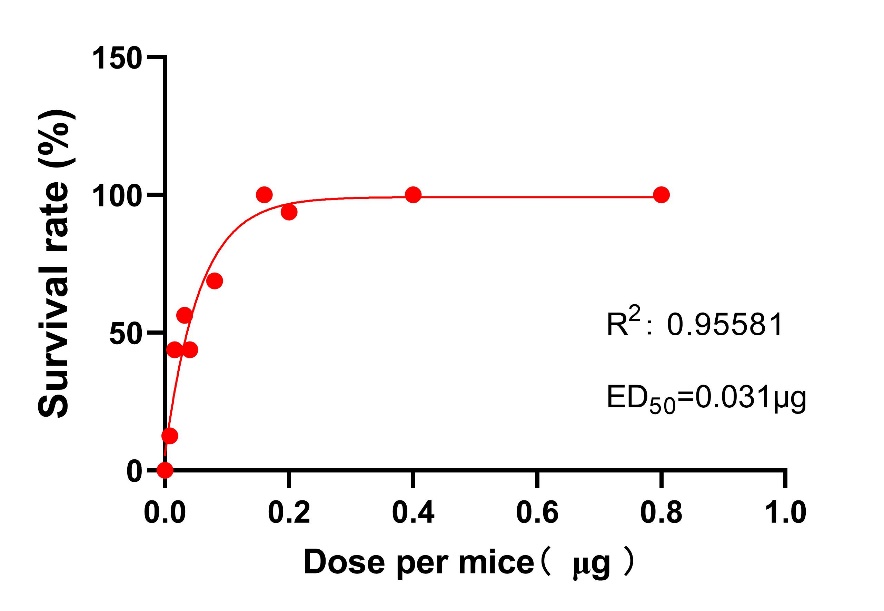


**Figure S2.** Dose-survival rate curve of the protective effect of RV021 vaccination against live-virus challenge in mice.

**Table S1.** Potency of RV021 as measured using the NIH method.

| **Group** | **Dilution** | **Survival** | | **Accumulated** | | | **Result** | |
| --- | --- | --- | --- | --- | --- | --- | --- | --- |
|  |  | **Death** | **Live** | **Death** | **Live** | **Death Rate** | **Log ED_50_** | **Potency** |
| RV021  (50 μg/dose) | ×25 | 0 | 16 | 0 | 42 | 0% | 3.00 | >16.1 IU/dose |
|  | ×125 | 2 | 14 | 2 | 26 | 7.14% |  |  |
|  | ×625 | 3 | 12 | 5 | 12 | 29.41% |  |  |
| IRV^1^ | ×25 | 1 | 15 | 1 | 39 | 2.5% | 2.91 | >13.6 IU/dose |
|  | ×125 | 3 | 12 | 4 | 24 | 14.29% |  |  |
|  | ×625 | 4 | 12 | 8 | 12 | 40% |  |  |
| 9^th^ Std^2^ | ×25 | 0 | 16 | 0 | 34 | 0% | 2.55 | 11.4 IU/mL |
|  | ×125 | 6 | 10 | 6 | 18 | 25% |  |  |
|  | ×625 | 8 | 8 | 14 | 8 | 63.6% |  |  |

^1^ Inactivated rabies vaccine for human use.

^2^ 9^th^ Chinese National Standard for human Rabies vaccines potency.
